# Effect of Low-Intensity Pulsed Ultrasound on Macrophage Properties and Fibrosis in the Infrapatellar Fat Pad in a Carrageenan-Induced Knee Osteoarthritis Rat Model

**DOI:** 10.7759/cureus.59246

**Published:** 2024-04-29

**Authors:** Takashi Kitagawa, Hirohisa Kawahata, Shintarou Kudo

**Affiliations:** 1 Graduate School of Health Sciences, Morinomiya University of Medical Sciences, Osaka, JPN; 2 Department of Rehabilitation, Higashiosaka Hospital, Osaka, JPN; 3 Inclusive Medical Sciences Research Institute, Morinomiya University of Medical Sciences, Osaka, JPN

**Keywords:** low-intensity pulsed ultrasound, macrophage, knee osteoarthritis, infrapatellar fat pad, fibrosis

## Abstract

Background: In the progression of knee osteoarthritis (KOA), fibrosis of the infrapatellar fat pad (IFP) is a key pathological change. Low-intensity pulsed ultrasound (LIPUS) inhibits IFP fibrosis by decreasing the gene expression and activity of hypoxia-inducible factor (HIF-1α), which is a protein involved in IFP fibrosis in KOA rat models. On the other hand, macrophages play an important role in the progression of fibrosis in various tissues, and LIPUS irradiation suppresses macrophage infiltration and inflammatory cytokine secretion. However, whether LIPUS suppresses macrophage polarity and IFP fibrosis in KOA remains unclear. Therefore, we investigated the effect of LIPUS on macrophage polarity and IFP fibrosis.

Materials and methods: A KOA model was created by injecting carrageenin into the bilateral knee joints of Wistar rats (eight weeks old). Tissues were harvested over time for histological and molecular biological analysis. The KOA model was also subjected to LIPUS irradiation for two weeks following the injection of carrageenin.

Results: RM-4-positive cells were widely distributed in IFP two weeks after carrageenin administration, but M2 macrophages were significantly increased, and the Sirius red area was decreased in the LIPUS-irradiated group compared with those in the non-irradiated group. The gene expression of M1 macrophage markers was significantly decreased and that of M2 macrophage markers was significantly increased in the LIPUS-irradiated group. The expression of transforming growth factor-β (TGF-β) and type 1 collagen was also significantly decreased.

Conclusion: These results suggest that LIPUS may serve as a novel approach for the treatment of KOA through its effect on M1 macrophages and suppression of TGF-β expression.

## Introduction

Knee osteoarthritis (KOA) is a chronic disease caused primarily by degenerative changes in articular cartilage, leading to symptoms such as limited knee joint range of motion, joint pain, and gait disturbance. Along with disease progression, there is a decline in activities of daily living and quality of life, resulting in frailty and bedriddenness [1].

However, degenerative changes in articular cartilage, which is considered the main pathology of KOA, may not align with symptoms. Instead, synovitis correlates with pain severity [2], suggesting that the pathogenesis primarily stems from synovial tissue lesions. Recent attention has focused on pathological changes in the infrapatellar fat pad (IFP) and their role in KOA symptoms and pathological progression [3].

The IFP is a fibrous fatty tissue situated in the knee joint space bordered by the patellar tendon, proximal tibia, and femoral condyle at the inferior border of the patella [4,5]. It attaches to the superior border of the patella and the internal and external patellar glenoid. Together with synovial tissue, the IFP facilitates smooth knee joint motion function [5]. During normal knee joint motion, it adjusts contact pressure by morphological changes and buffers the joint load [6]. However, in patients with KOA, the IFP appears as a low-signal image on MRI, exhibiting fibrosis [7]. This suggests that changes in mechanical properties diminish its capacity to buffer against joint loading [8,9]. These findings highlight IFP fibrosis as a significant factor in knee joint functional impairment, emphasizing the importance of suppressing pathological IFP changes for maintaining and improving joint function in KOA treatment.

This study investigated the mechanism of IFP fibrosis in a KOA rat model and identified its strong involvement with transcriptional activity of hypoxia-inducible factor (HIF-1α) [10]. However, inflammation may serve as a starting point alongside HIF-1α activity, and the role of macrophages has been attracting attention in recent years. Furthermore, in the IFP of patients with KOA, infiltration of lymphocytes and macrophages is observed, with increased expression of interleukin-6 (IL-6) and tumor necrosis factor-α (TNF-α) [11,12]. Additionally, in the IFP of KOA, there is elevated secretion of IL-6, interleukin-8 (IL-8), prostaglandin F2a (PGF2α), and TNF-α, followed by synovial fibrosis of the synovial membrane [13]. To inhibit IFP fibrosis in KOA, it is crucial to understand the dynamics of cells and molecules related to inflammation since macrophage polarization is considered to play an essential role in developing fibrosis in organs and tissues [14]. Similarly, the role of macrophages in IFP fibrosis necessitates further investigation.

Macrophages differentiate into functionally distinct phenotypes in response to pathological stimuli. These are classified into M1 and M2 macrophages that act primarily in inflammatory and anti-inflammatory manners, respectively, and engage in tissue repair [15]. M1 macrophages secrete proinflammatory cytokines TNF-α and interleukin-1β (IL-1β), which promote angiogenesis, fibroblast proliferation, and extracellular matrix synthesis [16,17]. Changes in M1 macrophage/M2 macrophage distribution in myocardial infarction nests and renal injury are associated with condition severity and fibrosis development [18,19]. Therefore, macrophage polarization likely plays a key role in IFP fibrosis in KOA, emphasizing the importance of inflammation control to reduce IFP fibrosis.

Guidelines recommend treating KOA with physical therapy, including orthotics, exercise therapy, and medication [20-22]. Among these, ultrasound therapy, a type of physical therapy, has been proven effective for KOA pain [23], increasing range of motion [24], reducing swelling and pain, and improving function [25] via thermal effect and intense vibration stimulation [26]. The mechanism of low-intensity pulsed ultrasound (LIPUS) irradiation has also been reported to decrease macrophage infiltration into the synovial tissue and reduce inflammatory cytokines secretion [26,27]. Furthermore, LIPUS has been recently reported to affect macrophage polarization in muscle repair and renal disease [28,29]. However, it remains unclear whether LIPUS alters macrophage polarization in the IFP of KOA, potentially reducing IFP fibrosis. This study reports the effects of LIPUS irradiation on the IFP in KOA model rats on macrophage polarization and fibrosis.

## Materials and methods

KOA model preparation and LIPUS irradiation

In this study, 36 male eight-week-old Wistar rats (180.8±13.9 g) were anesthetized with 1.5% isoflurane mixed with oxygen, and 50 μL of 0.5% carrageenin was injected into both knee joints to create a KOA model rat. These were randomly divided into two groups: (1) car group (18 rats), wherein carrageenin was administered without LIPUS irradiation; and (2) car+LIPUS group (18 rats), wherein carrageenin was administered with LIPUS irradiation. LIPUS was delivered every other day for 15 minutes per day, four times a week, at a frequency of 3 MHz and an output of 120 mW/cm² [27]. The control group was the saline group (18 animals), receiving saline in both knee joint cavities. In both groups, knee joints were harvested at one and two weeks after car administration (mentioning anesthesia), and samples were isolated for analysis via real-time polymerase chain reaction (PCR) and histological studies. This experiment was approved by the Morinomiya Medical University Animal Experimentation Ethics Committee and was conducted in accordance with the Morinomiya Medical University Animal Experimentation Guidelines (Research Ethics Approval Number: 2022A002).

Histological analysis

The collected knee joints were fixed with 4% paraformaldehyde in 0.1 M phosphate buffer (pH 7.4), demineralized with Mohs’ solution, and paraffin-embedded using standard techniques. The sections were then thinned to 5 µm in the sagittal plane and stained with hematoxylin-eosin (HE) solution using standard methods. Additionally, the condition of the articular cartilage was assessed based on the scoring method recommended by the International Osteoarthritis Research Society (OARSI) using toluidine blue staining [30]. Fibrosis was evaluated by measuring collagen I and III staining areas with the picrosirius red stain kit (Sirius red (SR) stain; Polysciences, Inc.; Warrington, PA) according to the manufacturer’s protocol. The area within the dotted square in the IFP was set as the measurement area, and the area of each stain was determined. The stained area was measured using ImageJ (version 1.48; National Institutes of Health, Stapleton, NY). To confirm myofibroblast distribution, immunohistological staining (IHC) with anti-α-smooth muscle actin (α-SMA) antibody (Abcam, Cambridge, MA) was conducted. IHC was blocked with 0.5% blocking reagent (Roche Applied Science, Indianapolis, IN, USA) - TBTS - after antigen inactivation and endogenous perkiositase removal with 0.02% proteinase K (Wako, Tokyo, Japan) following tissue section deparaffinization. After blocking, the diluted antibody (1:100) was allowed to react for one hour at room temperature. After washing, the antibody was reacted with Histofine-synthesized mouse MAX-PO (rat) (Nichirei Bioscience, Tokyo, Japan) as a secondary antibody for 30 minutes. Color reaction was then performed in 0.3% DAB solution containing 3% hydrogen peroxide and sealed.

Macrophage distribution and M1/M2 ratio

IHC verified macrophage distribution using an anti-RM-4 antibody (Medical Chemistry Pharmaceutical Inc., Hokkaido, Japan) against all macrophages, an anti-CD80 antibody (Proteintech, IL, USA) against M1 macrophages, and an anti-CD206 antibody (Proteintech) against M2 macrophages. IHC was performed using anti-CD80 antibody (Proteintech) and anti-CD206 antibody (Proteintech) against M1 and M2 macrophages, respectively. Image analysis was performed using ImageJ (version 1.48; National Institutes of Health, Stapleton, NY) to measure the area where positive cells for each antibody were distributed, and results were compared and examined in each group.

Gene expression of macrophage markers and fibrosis-related factors

Total RNA was extracted from the knee capsule excluding the cartilage and meniscus. Excised tissues were homogenized in cold phosphate-buffered saline (PBS) and centrifuged at 20,000 × g for 15 min at 4°C. The total RNA of tissue samples was extracted using ISOGEN II (NIPPON GENE, Toyama, Japan), re-suspended in PBS, and assessed by spectrophotometry to determine their purity. Only samples with a ratio of spectrophotometric absorbance at 260 nm to that at 280 nm (A260/A280) in the range of 1.9-2.1 were used. Complementary DNA was synthesized using an iScript cDNA Synthesis Kit (Bio-Rad Laboratories, Hercules, CA). Amplification reactions were performed with SsoFast EvaGreen Supermix (Bio-Rad Laboratories, Hercules, CA), with 100 μM primers and 1 μL cDNA in a final volume of 20 μL. Amplification reactions were carried out in a MiniOpticon Real-Time PCR Detection System (Bio-Rad Laboratories, Hercules, CA), according to the manufacturer’s instructions (one min at 95°C, followed by 40 cycles each of one sec at 95°C and five seconds at 61-65°C) with each primer. The expression level of HRPT as a housekeeping gene was used as the internal control, and the comparative Ct method (2ΔΔCt) was used to quantify gene expression. The relative expression by delta Ct is calculated by subtracting the crossing point cycle for the housekeeping gene from those for the genes analyzed. The oligonucleotides were synthesized using Gene Design (Suita, Japan). The primer sequences used were obtained from previous studies [10,31-33] and are listed in Table 1.

**Table 1:** Primers designed for real-time PCR.

Name	Sense primer (5’-3’)	Antisense primer (5’-3’)	Ref.
IL-1β	GGGTTCCATGGTGAAGTCAAC	CACCTCTCAAGCAGAGCACAG	[31]
iNOS	AGCATCCACGCCAAGAACG	GTCTGGTTGCCTGGGAAAAT	[32]
IL-10	ACTGCTATGTTGCCTGCTCTTAC	CAGTAAGGAATCTGTCAGCAGTATG	[32]
Arg1	ATTGGCAAAGTGATGGAAGAGAC	CAAGACAAGGTCAACGCCAC	[32]
TGF-β	AGAAGTCACCCGCGTGCTAAT	CACTGCTTCCCGAATGTCTGA	[33]
Col 1a2	GCTTTGTGGATACGCGAACTC	CCAGCATTGGCATGTTGCT	[33]
HRPT	TGTTTGTGTCATCAGCGAAAGTG	ATTCAACTTGCCGCTGTCTTTTA	[10]

Statistical analysis

Statistical analyses were performed using EZR (version 1.61; R Foundation for Statistical Computing, Vienna, Austria). The Shapiro-Wilk test was used to test normality. For two-group comparisons, data distribution was assessed, and an unpaired t-test was used. For analyses involving three or more groups, data distribution was initially examined using the Tukey-Kramer post-hoc test after one-way ANOVA or the Steel-Dwass post-hoc test after Kruskal-Wallis. The significance level was set at less than 5%.

## Results

Fibrosis of IFP and distribution of macrophages after treatment with carrageenin

**Figure 1:**
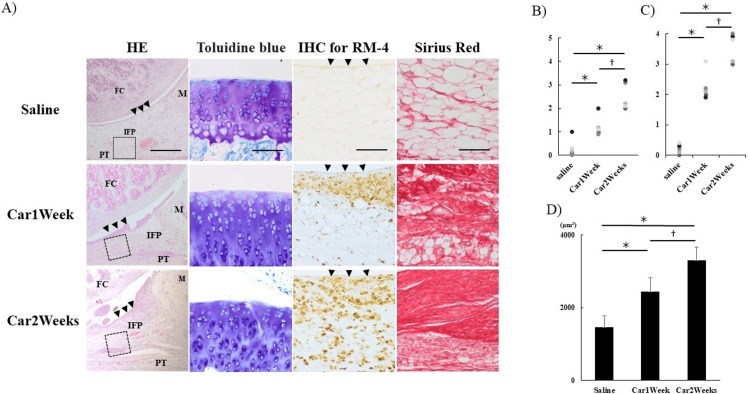
Histological analysis of IFP. (A) HE staining (left), toluidine blue staining (center left), IHC (RM-4) staining (center right), SR staining (right). Left: magnification ×40, scale bar 500 μm; (center left), magnification ×200, scale bar 100 μm; middle right: magnification ×200, scale bar 100 μm; right: magnification ×200, scale bar 100 μm; ▼ (arrowhead) indicates synovial endothelium. (B) OARSI score (articular cartilage), (C) OARSI score (synovium), (D) quantitative analysis of collagen content in synovium measured using ImageJ. *P<0.05, **P<0.01 (vs Saline), †P<0.05 (Car1Weeks vs Car2Weeks). n=5 per group. Data are presented as mean ± SD. HE: hematoxylin-eosin staining; SR: Sirius red staining; IHC: immunohistochemical staining; IFP: infrapatellar fat pad; FC: femoral condyle; M: meniscus; PT: patellar tendon; Saline: rats injected with saline only; Car1Week: knee joint after 1 week of carrageenin injection; Car 2 weeks: knee joint after 2 weeks of carrageenin injection

HE staining and toluidine blue staining showed progressive cellular infiltration of IFP and fibrous tissue growth over time in the car group compared to those in the saline group. OARSI scores in the synovium and articular cartilage increased significantly. Synovial OARSI score: The results of the Kruskal-Wallis test showed a statistically significant difference between groups (χ²(2) = 12.895, P<0.05); the results of the Steel-Dwass test showed a significant difference between all groups (P<0.05). Articular cartilage OARSI score: ANOVA results showed statistically significant differences between groups (F(2, 12) = 26.00, P<0.05; Figures 1A-1C). IHC revealed a broader distribution of RM-4-positive cells in the car group than in the saline group, and the distribution increased over time (Figure 1A). SR staining indicated the presence of collagen fibers in the IFP at both one and two weeks in the car group, with significant fibrosis development at two weeks (SR area comparison (saline vs Car1Week vs Car2Weeks) of ANOVA results showed statistically significant differences between groups (F(2, 12) = 27.44, P<0.05). Post-hoc test results showed significant differences between all groups (P<0.05, Figure 1D)).

Effect of LIPUS on macrophage traits and fibrosis

**Figure 2:**
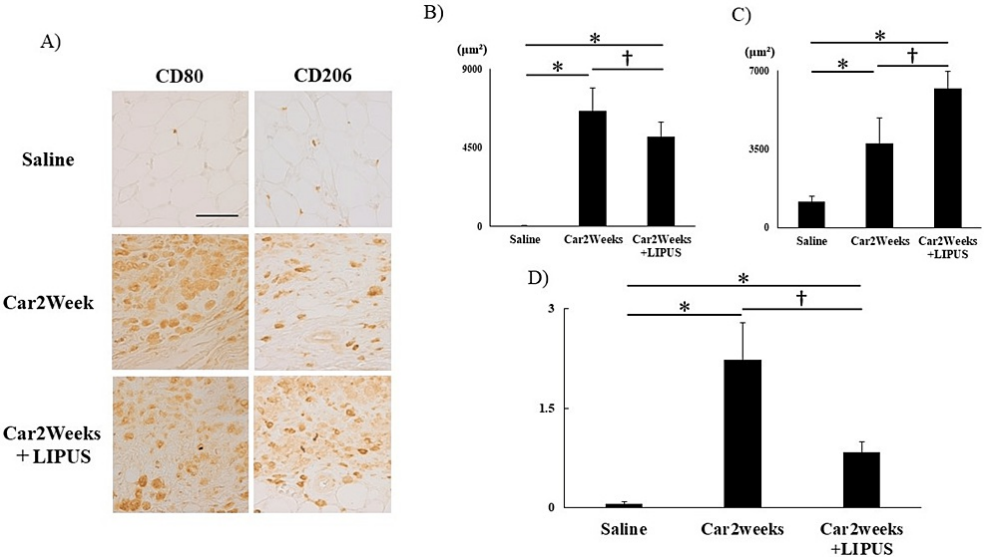
Histological analysis of macrophage distribution in IFP. (A) IHC against CD80-positive cells (left) and CD206-positive cells (right). Panel: magnification ×400, scale bar 50 μm. (B) Quantitative analysis of CD80-positive cells in IFP measured using ImageJ. (C) Quantitative analysis of CD206-positive cells within IFP measured using ImageJ. (D) Comparison of the ratio of M1 macrophage-positive cells/M2 macrophage-positive cells. *P<0.05 (vs saline), †P<0.05 (Car2Weeks vs Car2Weeks＋LIPUS). n=5 per group. Data are presented as mean ± SD. IHC: immunohistochemical staining; Saline: rats injected with saline only; Car2weeks: knee joint after 2 weeks of carrageenin injection; Car2Weeks+LIPUS: knee joint irradiated with LIPUS for 2 weeks after carrageenin injection

At two weeks after carrageenin administration, the car+LIPUS group exhibited a significant decrease in the OARSI score compared to the car group (P<0.05, data not shown). The number of CD80-positive cells per unit area was significantly lower in the car+LIPUS group than in the car group, and the distribution of CD206-positive cells was significantly wider in the LIPUS-irradiated group (CD80 area comparison (saline vs Car2Weeks vs Car2Weeks+LIPUS)): Kruskal-Wallis test results showed statistically significant differences between groups (χ²(2) = 12.02; P<0.05), whereas Steel-Dwass test results showed significant differences between all groups (P<0.05). CD206 area comparison (saline vs Car2Weeks vs Car2Weeks+LIPUS): ANOVA results showed a statistically significant difference between groups (F(2, 12) = 42.94, P<0.05; Figures 2A-2C). The ratio of CD80-/CD206-positive cells was significantly lower in the car+LIPUS group (CD80/CD206 area comparison (saline vs Car2Weeks vs Car2Weeks+LIPUS)). ANOVA results showed statistically significant differences between groups (F(2, 12) = 41.33; P<0.05; Figure 2D).

**Figure 3:**
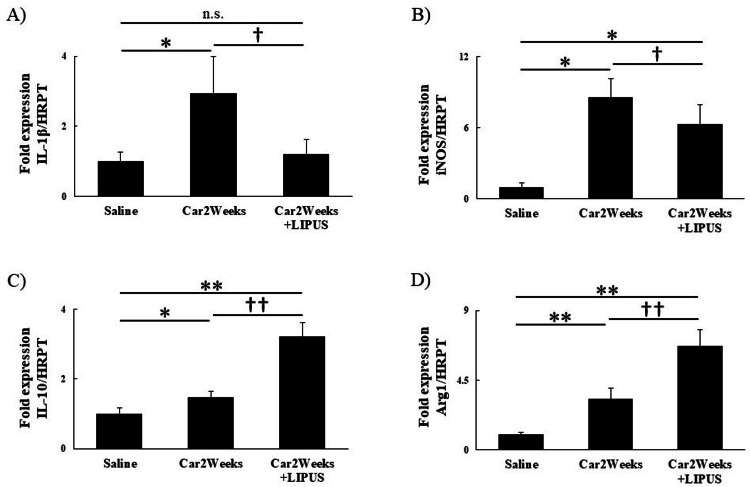
mRNA expression of IL-1β, iNOS, IL-10, and Arg1 in IFP. Relative mRNA expression of (A) IL-1β, (B) iNOS, (C) IL-10, and (D) Arg1. n.s: not significant. *P<0.05, **P<0.01 (vs Saline), †P<0.05, ††P<0.05 (Car2Weeks vs Car2Weeks+LIPUS). n=5 per group. Data are presented as mean ± SD. IL-1β: interleukin 1 beta; iNOS: inducible nitric oxide synthase; IL-10: interleukin 10; Arg1: Arginase 1; Saline: knee joints of rats injected with saline only; Car2weeks: knee joint after 2 weeks of carrageenin injection; Car2weeks+LIPUS: knee joint irradiated with LIPUS for 2 weeks after carrageenin injection

Furthermore, the gene expression of macrophage markers was significantly lower in the car+LIPUS group for M1 macrophage markers (IL-1β (saline vs Car2Weeks vs Car2Weeks+LIPUS)): The results of the Kruskal-Wallis test showed a statistically significant difference between the groups (χ²(2) = 7.52; P<0.05). The results of the Steel-Dwass test showed significant differences in the saline vs. carrageenin group and the carrageenin vs. LIPUS group (P<0.05); no significant difference was found in the saline vs. LIPUS group. iNOS (saline vs Car2Weeks vs Car2Weeks+LIPUS): The results of the Kruskal-Wallis test showed a statistically significant difference between the groups (χ²(2) = 11.58; P<0.05), whereas those of the Steel-Dwass test showed a significant difference between all groups (P<0.05; Figures 3A, 3B) and was significantly higher in the Car+LIPUS group for M2 macrophage markers (IL-10 (saline vs Car2Weeks vs Car2Weeks+LIPUS): The results of the Kruskal-Wallis test showed statistically significant differences between groups (χ²(2) = 12.02; P<0.05); the results of the Steel-Dwass test showed significant differences between all groups (P<0.05). Arg1 (saline vs Car2Weeks vs Car2Weeks+LIPUS): ANOVA results showed statistically significant differences between groups (F(2, 12) = 55.20, P<0.05). Post-hoc test results showed significant differences between all groups (P<0.05; Figures 3C, 3D).

**Figure 4:**
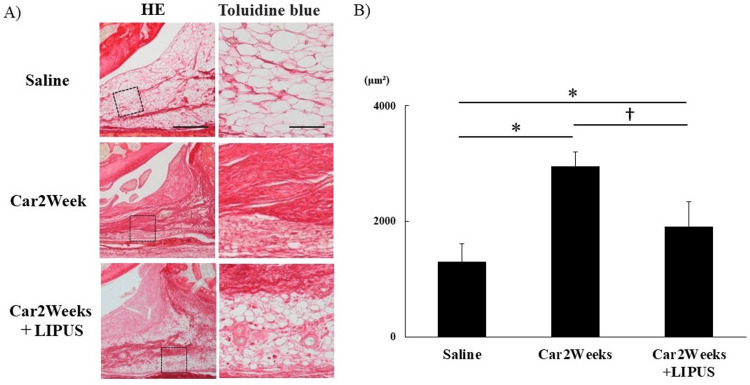
Histological analysis of the effect of LIPUS on IFP fibrosis assessed by SR staining. (A) SR staining. Left top and bottom: magnification ×40, scale bar 500 μm; right top and bottom: magnification ×200, scale bar 100 μm. (B) Quantitative analysis of collagen content in IFP measured using ImageJ. *P<0.05, **P<0.01 (vs Saline), †P<0.05, ††P<0.05 (Car2Weeks vs Car2Weeks+LIPUS). n=5 per group. Data are presented as mean ± SD. SR: Sirius Red staining; IFP: infrapatellar fat pad; Saline: rats injected with saline only; Car2weeks: knee joint after 2 weeks of carrageenin injection; Car2Weeks+LIPUS: knee joint exposed to LIPUS for 2 weeks after carrageenin injection

The inhibitory effect of LIPUS on fibrosis of the IFP was also examined. Histological analysis showed that the SR-stained area of an IFP treated with LIPUS for two weeks in the car+LIPUS group was significantly reduced compared to the car group (Figure 4A), indicating that LIPUS irradiation suppressed synovial fibrosis (SR staining comparison (saline vs Car2Weeks vs Car2Weeks+LIPUS): the Kruskal-Wallis test results showed statistically significant differences between groups (χ²(2) = 12.02; P<0.05), whereas the Steel-Dwass test results showed significant differences between all groups (P<0.05; Figure 4B).

**Figure 5:**
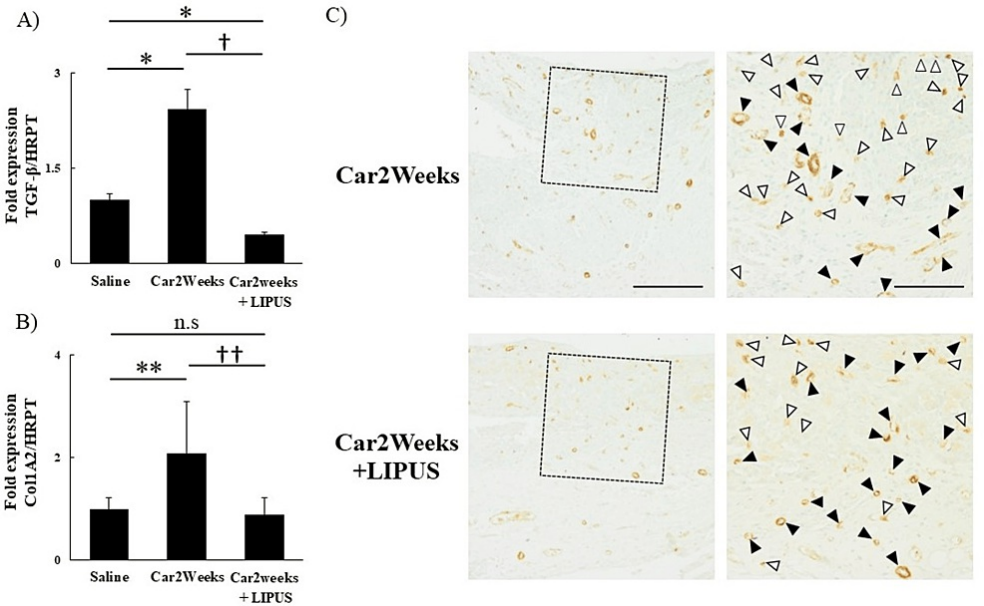
mRNA expression of TGF-β and type I collagen and distribution of α-SMA-positive cells in IFP. Relative mRNA expression of (A) TGF-β and (B) type I collagen. (C) IHC for α-SMA-positive cells. Left top and bottom: magnification ×100, scale bar 200 μm; right top and bottom: magnification ×200, scale bar 100 μm. n.s: Not significant. *P<0.05, **P<0.01 (vs Saline), †P<0.05, ††P<0.05 (Car2Weeks vs Car2Weeks+LIPUS). n=5 per group. Data are presented as mean ± SD. Black arrows indicate blood vessels; white arrows indicate α-SMA-positive cells. TGF-β: transforming growth factor-β; ColA2: alpha1 chain of type I collagen; Saline: knee joint injected with saline only; Saline, Car 2 weeks: knee joint after 2 weeks of carrageenin injection, Car2Weeks+LIPUS: knee joint after 2 weeks of LIPUS after carrageenin injection

In the car+LIPUS group, the fibrosis-associated genes, such as those that encode TGF-β and type 1 collagen, were also significantly decreased (TGF-β (saline vs Car2Weeks vs Car2Weeks+LIPUS)): The results of the Kruskal-Wallis test showed statistically significant differences between groups (χ²(2) = 12.50; P<0.05); the results of the Steel-Dwass test showed significant differences between all groups (P<0.05). Col1A2 (saline vs Car2Weeks vs Car2Weeks+LIPUS): The results of the Kruskal-Wallis test showed a statistically significant difference between the groups (χ²(2) = 9.78; P<0.05). The results of the Steel-Dwass test showed significant differences (P<0.05) in the saline vs. carrageenin group and the carrageenin group vs. LIPUS group; on the other hand, no significant difference was found in the saline vs. LIPUS group (Figures 5A, 5B). Additionally, α-SMA-positive cells were distributed in a localized area in the car+LIPUS group (Figure 5C).

## Discussion

The IFP is primarily composed of adipocytes, as well as fibroblasts, monocytes, lymphocytes, macrophages, and other cells producing extracellular organelles [34]. It serves as a tissue with a buffering effect on smooth joint motion and mechanical loading [5]. However, in the development of KOA, inflammation and fibrosis occur in the IFP, leading to symptoms such as pain and limited range of motion that are considered to contribute to KOA progression [3,11]. Therefore, a comprehensive understanding of the pathological changes in IFP is necessary to halt KOA progression.

Previously, the authors demonstrated that HIF-1α activation is involved in IFP fibrosis via carrageenin administration [10]. However, in the present study, the extensive distribution of RM-4-positive cells in the IFP with carrageenin administration indicates a significant role of macrophages in IFP fibrosis. In the IFP, adipocytes secrete PGF2α and induce synovitis [13]. Macrophages are also activated by inflammatory cytokines from adipocytes and other immune cells, which secrete inflammatory cytokines and matrix metalloproteinases [14]. This induces fibrosis of synovial tissue and cartilage degradation [34-36]. In KOA, extensive activated macrophages are present in the synovial tissue along with the IFP. Likewise, an increase in activated macrophages is significantly associated with severity of knee joint pain and KOA severity based on radiographic findings [37]. Therefore, an increase in macrophages and activation of macrophages may play a significant role in the pathological progression of KOA [38].

Macrophages can be classified into two types based on phenotype: pro-inflammatory M1 macrophages and anti-inflammatory M2 macrophages [39,40]. M1 macrophages secrete proinflammatory cytokines such as TNF-α, IL-1β, and IL-6, which have proinflammatory effects. In the IFP/synovium complex of KOA, prolonged M1 macrophage dominance also leads to fibrosis of IFP [12]. Meanwhile, suppression of M1 macrophage activity is reported to reduce fibrosis [41-43]. Therefore, in the present study, the predominance of M1 macrophages is considered to cause fibrosis of IFP after carrageenin administration, as most macrophages distributed in IFP were CD80-positive cells.

TGF-β, which is secreted by fibroblasts and macrophages, regulates cell differentiation and proliferation, and is considered to promote tissue repair such as wound healing [44]. However, it is also strongly implicated in tissue fibrosis [45] and associated with pathological fibrosis, like renal fibrosis and cirrhosis of the liver [46,47].

Notably, carrageenin treatment increased TGF-β expression in the IFP in this study. TGF-β is known to contribute to KOA pathogenesis, articular cartilage degeneration, and synovial fibrosis [48,49]. It is reported to be secreted by M2 macrophages [50]. However, TGF-β is also secreted by fibroblasts, endothelial cells, and lymphocytes, in addition to macrophages [45]. TGF-β is expressed in proliferating synovial fibroblasts in fibrotic synovial tissue in rheumatoid arthritis and joint contractures [51-53]. Moreover, TGF-β induces myofibroblast differentiation into fibroblasts and promotes fibrosis [54,55]. Despite not identifying any cells expressing TGF-β in the IFP, the increased TGF-β expression and wide distribution of α-SMA-positive cells after carrageenin treatment suggest their significant role in fibrosis development.

The finding that LIPUS irradiation reduced fibrosis of the IFP in this study is a significant result in treating KOA. However, the underlying mechanism remains unclear. In our previous analysis focusing on HIF-1 as a factor associated with fibrosis, it was revealed that LIPUS suppressed HIF-1 activity, thereby reducing IFP fibrosis [10]. Recently, it has been pointed out that HIF-1 and macrophage polarization are related [56]. We also investigated the effect of LIPUS on macrophage polarization and found that M1 macrophage distribution decreased, and M2 macrophages predominated in the LIPUS-exposed group. The gene expression of IL-1β and iNOS, which are markers of M1 macrophages, was decreased. Meanwhile, that of IL-10 and Arg-1, which are markers of M2 macrophages, was increased by LIPUS irradiation. This indicates that LIPUS has an effect on macrophage polarization in addition to suppressing HIF-1α activity.

As for the effects of LIPUS on macrophage polarization, studies have shown that LIPUS irradiation suppresses inflammatory responses and fibrosis in drug-induced renal injury [29]. Additionally, it promotes skeletal muscle regeneration by enhancing M2-shifted macrophage polarization during the skeletal muscle repair process [28,29]. Therefore, it is thought that LIPUS affected macrophage polarization in the IFP, resulting in a predominance of M2 macrophages and suppression of fibrosis.

Despite not examining the direct mechanism by which LIPUS affects macrophage polarization, it has been reported that LIPUS regulates macrophage gene expression and suppresses cell death. This results in a decreased release of heat shock protein and an increase in M2 macrophages. Additionally, LIPUS has been reported to increase the number of macrophages [29], suggesting a mechanism that involves the regulation of macrophage function through these effects.

The mechanism by which LIPUS directly affects the expression of TGF-β and other genes has not been investigated in this study; hence, further investigation is needed. However, it has been reported that LIPUS has the following effects: (1) decreases TGF-β expression in articular cartilage [57], (2) inhibits TGF-β-induced fibrosis in cultured fibroblast-like synoviocytes [58], (3) reduces osteoclast activity by suppressing TGF-β1/Smad3 signaling [59], and (4) reduces subchondral bone resorption by decreasing osteoclast activity via TGF-β1/Smad3 signal suppression [59]. LIPUS irradiation still reduces TGF-β gene expression and localizes the distribution of α-SMA-positive cells. Therefore, it is suggested that LIPUS also has a direct effect on TGF-β function, thereby reducing fibrosis.

Although this study showed that macrophages are involved in the development of IFP fibrosis in KOA and that LIPUS affects macrophage polarization, it has several limitations. One of these is the technical limitation regarding IFP sample collection: synovial tissue could not be completely removed from the IFP because of its continuous histological connection with the surrounding tissue. Therefore, the stained area and gene expression may also reflect the responses of the synovial tissue. Second, we used carrageenin to induce synovitis. Since the involvement of mechanical stress in the etiology of KOA has been demonstrated before, the relationship between HIF-1α and IFP fibrosis in OA models with unstable joints such as the medial meniscus or OA models that underwent ligamentectomy must be investigated further. Another limitation of our study is that we did not examine the direct mechanism by which LIPUS affects macrophage polarization despite the possibility that LIPUS may directly modulate macrophage function as suggested by studies reporting that LIPUS increases the number of M2 macrophages by regulating macrophage gene expression, decreasing the release of heat shock protein, and inhibiting cell death [29]. Furthermore, the mechanism by which LIPUS directly affects the expression of genes such as TGF-β was not investigated in this study even though it has been reported that LIPUS reduces TGF-β expression in articular cartilage [57], inhibits TGF-β-induced fibrosis in cultured fibroblast-like synoviocytes [58], reduces osteoclast activity by suppressing TGF-β1/Smad3 signaling, and reduces subchondral bone resorption by reducing osteoclast activity. These findings suggest that LIPUS may directly affect both the gene expression and function of TGF-β and must hence be investigated in detail. Finally, the effect of LIPUS on IFP fibrosis was evaluated only two weeks after LIPUS irradiation. In other words, the lack of data after one week is a limitation of this study.

## Conclusions

The results of this study indicate that carrageenin administration promotes fibrosis by inducing the infiltration of M1 macrophages into the IFP and increasing TGF-β expression. In contrast, LIPUS irradiation significantly increased the number of M2 macrophages and suppressed TGF-β expression, thus inhibiting fibrosis of the IFP. These results suggest that macrophage polarity is involved in IFP fibrosis in KOA and that LIPUS inhibits IFP fibrosis by altering macrophage polarity. These results are useful for developing treatment strategies for KOA in the field of physical therapy.
